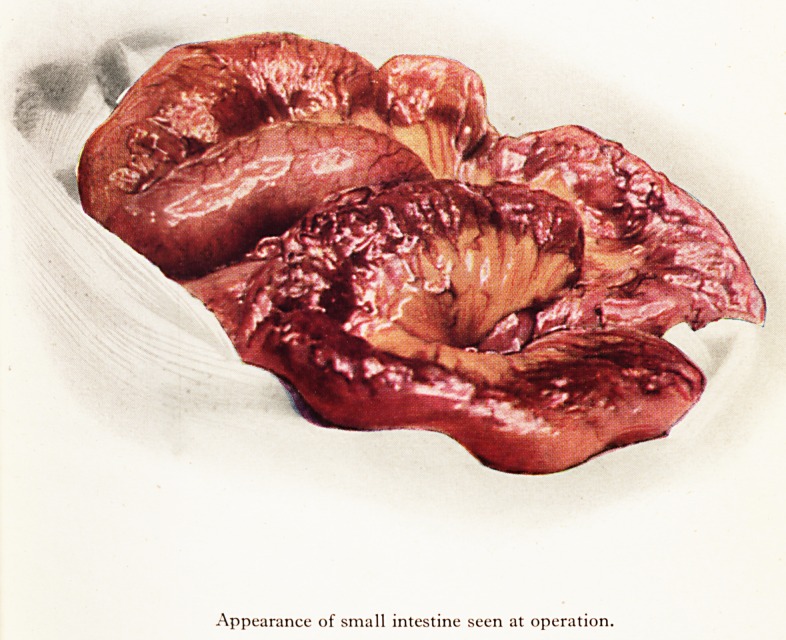# Diffuse Angiomatosis of the Small Intestine Causing Melaena

**Published:** 1954

**Authors:** John A. Vere Nicoll

**Affiliations:** Yeovil


### DIFFUSE ANGIOMATOSIS OF THE SMALL INTESTINE CAUSING MELAENA

BY

JOHN A. VERE NICOLL, F.R.C.S., D.A.

*Yeovil*

In a recent paper Shepherd (1953) discussed the various types of angiomatous disorders of the gastro-intestinal tract and remarked that few examples had been reported in this country. The following case is therefore interesting, especially because the telangiectasia was more extensive than in previously described patients in whom operation was possible.

### CASE REPORT

A married woman, aged thirty-eight and with five children, was urgently admitted to hospital because of extreme tiredness and weakness following a severe melaena on 6th April, 1953. Ten days previously she had returned home after spending fifteen years in Northern Rhodesia. She had felt tired since the birth of her third child six years ago, but all her pregnancies and deliveries had been normal. Her periods were regular, lasting three to four days, fairly heavy, every twenty-five days. Her appetite was fair and she had had no indigestion. She had never suffered from nose-bleeding. There had been severe loss of weight during the past few weeks with increasing weakness and tiredness. There was no history of malaria, dysentery, hookworm infection, or any other serious disease. Recently she had been constipated and had taken " Agarol ". Three weeks before admission after taking cascara she first noticed that her motions were very dark.

On her arrival in England ten days before admission, her friends remarked upon her pallor and she noticed it herself. Three days before admission she passed a dead round-worm, and later felt very tired and giddy. The day before admission she felt so weak that she was unable to get out of bed, but she had had no pain.

*Family history:* Mother alive and well. Father died of carcinoma of the bowel. All her children are well. There was no history of bleeding in any other member of the family.

*Condition on examination:* A co-operative and intelligent young woman: T.98; P.90 regular; R.24; B.P. 100/60. There was no rash, or enlarged glands. She looked very pale and anaemic. Heart and lungs normal. Breasts normal. Tongue furred—teeth and throat healthy. Abdomen—no abnormality detected on palpitation. P.R.—the rectum was empty and the cervix felt normal. C.N.S.—normal.

| Blood picture: | | |
|---|---|---|
| | R.B.C.s | = 3·2 millions per c.mm. |
| | Hb (Haldane) | = 44 per cent. or 6·1 gm. per cent. |
| | C.I. | = 0·69. |
| | W.B.C.s | = 11,000 per c.mm. |
| | Polys. | = 80 per cent. or 8,800 per c.mm.. |
| | Lymphs. | = 19 per cent. or 2,090 per c.mm. |
| | Monos. | = 1 per cent. or 110 per c.mm. |

Film: R.B.C.s show anisocytosis, poikilocytosis, and hypochromasia.

Blood group: " A "; Rh negative.

No worms, ova or cysts were found in the stools. On 8.4.53 she was given a blood transfusion of four pints. On 10.4.53 her haemoglobin was only 48 per cent. A barium meal showed no abnormality of the stomach or duodenum. On 12.4.53 she was very weak an[illegible] giddy when sitting up, and because she was obviously still bleeding a laparotomy w[illegible] decided upon. The possible diagnoses being considered after ruling out peptic ulcer, we[illegible] hookworm infestation or Meckel's diverticulum. She was given five pints of blood befor[illegible] during and after operation, making a total of nine pints.

12.4.53. OPERATION under pentothal, curare, gas and oxygen and ether anaesthes[illegible] A right paramedian incision was made. Immediately, dilated loops of small intesti[illegible] full of fresh and altered blood were seen. The stomach and duodenum were normal a[illegible] did not contain blood; but the jejunum presented a most remarkable appearance. Bloo[illegible] vessels of varying sizes were interlocked and woven in haphazard and tortuous patter[illegible] all over the outside of the gut involving at least half of the entire length of the sm[illegible] intestine. The first part of the jejunum was opened, but no obvious internal enlargeme[illegible] of vessels and no hookworms could be seen here or in the duodenum. No Meckel[illegible] diverticulum was found and the rest of the bowel and viscera was normal [*Plate X*].

To remove so large a segment of gut in this patient was thought unjustifiable. It w[illegible] decided to close the abdomen and try to arrest the bleeding by conservative methods, a[illegible] to continue with blood transfusion. The next day the patient was better and the haem[illegible] globin was 64 per cent. She ate well and did not feel giddy. A full second-stage gast[illegible] diet with added vitamins was given. On 16.4.53 the haemoglobin was 52 per cent. [illegible] two pints of blood were given. On 18.4.53 the haemoglobin was 66 per cent. and [illegible] patient felt well. An enema, however, still produced a melaena stool. The haemoglob[illegible] was 64 per cent. on 20.4.53, 60 per cent. on 24.4.53 and 50 per cent. on 25.4.53. A furth[illegible] blood transfusion of two pints was given, but on 26.4.53 the haemoglobin was still o[illegible] 50 per cent. despite transfusion the previous day, so it was decided to resect the wh[illegible] affected small intestine. Two pints of blood were given before, one pint during and thr[illegible] pints after the operation.

SECOND OPERATION: The abdomen was opened through the original paramedian sc[illegible] Adhesions were present which had to be separated carefully. The length of affect[illegible] segment and of the whole of the small intestine was then carefully measured, and it w[illegible] found that about seven feet of intestine had to be removed, leaving about three and a h[illegible] feet of jejunum proximally and five feet of ileum distally, the whole length being consid[illegible] ably less than the twenty-three feet usually described. After resection and end-to-e[illegible] anastomosis, the abdomen was closed. On opening the resected segment of gut no [illegible] larged vessels were seen but the mucous membrane had a red blush throughout, and [illegible] seemed that there had been steady oozing from all this mucous membrane.

*Pathological report*:

"Specimen—5 feet of small intestine opened. No macroscopic abnormality noted ap[illegible] from the blood vessels in the serous coat. These are apparently greatly increased [illegible] number and are tortuous. Diffuse telangiectasia of small intestine."

Subsequent progress was rapid and uneventful, and the haemoglobin climb[illegible] steadily to normal. Five months after the operation the patient had gained [illegible] stone in weight, and was very well. There were no symptoms suggesting i[illegible] paired fat absorption, and no signs of avitaminosis.

### DISCUSSION

It was difficult to decide upon the correct treatment for this patient. At t[illegible] first operation removal of so large a portion of small intestine was thoug[illegible] inadvisable, especially since spontaneous regression of individual haemorrha[illegible] lesions may occur (Shepherd, 1953). When, however, bleeding persisted, [illegible] section was clearly indicated and seemed to offer hope of complete cure, si[illegible] removal of as much as half the small intestine is not followed by serious me[illegible] bolic disturbance (Croot, 1952).

PLATE X

Appearance of small intestine seen at operation.

A diagnosis that was considered was hereditary telangiectasia (Osler-Rendu disease). But a meticulous examination of the patient revealed only one capillary telangiectasis over the inner side of one ankle. This had become enlarged and conspicuous during the patient's pregnancies. No other telangiectasis was found. Moreover, while a large proportion of patients with Osler-Rendu disease give a family history of the disorder (Shepherd, 1953), none was obtained in our patient. This case in fact is similar to another recently described as diffuse angiomatosis of the small intestine (Richardson and Flatt, 1953).

### REFERENCES

Shepherd, John A. (1953). *Brit. J. Surg.*, **40**, 409.

Croot, H. J. (1952), *Brit. med. J.*, **1**, 195.

Richardson, J. E., and Flatt, A. E. (1953). *Brit. med. J.*, **2**, 311.

## Figures and Tables

**Figure f1:**